# Pathogenicity and Host Range of *Pythium kashmirense*—A Soil-Borne Oomycete Recently Discovered in the UK

**DOI:** 10.3390/jof7060479

**Published:** 2021-06-12

**Authors:** Clara Benavent-Celma, Alexandra Puertolas, Debbie McLaggan, Pieter van West, Steve Woodward

**Affiliations:** 1 Department of Plant and Soil Science, School of Biological Sciences, University of Aberdeen, Aberdeen AB24 3UU, UK; clara.benaventcelma@abdn.ac.uk; 2 International Centre for Aquaculture Research and Development (ICARD), Aberdeen Oomycete Laboratory, Institute of Medical Sciences, University of Aberdeen, Foresterhill, Aberdeen AB25 2ZD, UK; d.mclaggan@abdn.ac.uk (D.M.); p.vanwest@abdn.ac.uk (P.v.W.); 3 ANSES, Laboratoire de la Santé des Végétaux–Unité de Mycologie, Domaine de Pixérécourt–Bât. E, CS 40009, F-54220 Malzéville, France; alexandra.puertolas@anses.fr

**Keywords:** *Pythium*, ornamental plants, pathogenicity, host range, soil-borne oomycete, root rot

## Abstract

During a survey of oomycetes in ornamental plants carried out at the University of Aberdeen in 2014–2015, *Pythium kashmirense* was isolated from a specimen of *Viburnum plicatum* ‘Lanarth’, the first report of this oomycete in the UK (and in Europe). Pathogenicity of a *Py. kashmirense* isolate was examined using a range of plant species. Inoculations were carried out under controlled conditions in the absence of other *Pythium* and *Phytophthora* species, on *Glycine max* (soya bean), *Phaseolus vulgaris* (common bean), *Lupinus angustifolius* (blue lupin), *Cucumis sativa* (cucumber) and *Viburnum opulus*. The majority of inoculations caused pre-emergence damping-off, as well as seed rot and root rot. In the in vitro assays, germination rates (%) of soya bean and blue lupin seeds were less than 50%; in the in vivo inoculations on plants, over 50% of soya bean, blue lupin and common bean plants died; in contrast, cucumber plants showed lower susceptibility in pathogenicity tests, with an approximately 80% germination rate in in vitro tests, and 25% dead plants in the in planta inoculations. Inoculations carried out on root systems of *Viburnum opulus* caused severe necrosis and root rot. Little research was previously conducted on pathogenicity of *Py. kashmirense* and its relationship with losses in crop yield and quality. The present study showed varying virulence on the different plant species tested after inoculation with *Py. kashmirense*. Despite the lack of clear host specialization, infection by *Py. kashmirense* decreased seedling survival and health of plants in a range of important agricultural and ornamental plant species.

## 1. Introduction

The genus *Pythium*, first established by Pringsteim in 1858, includes many plant pathogens, with global distributions in various habitats. These species cause pre- and post-emergence damping-off of seedlings, as well as root rot, crown rot and blight in a wide range of plant hosts [1]. Currently, over 150 species of *Pythium* are formally recognised, of which 41 species have been described since 2000 [2,3,4,5]. One of the most significant members in this genus is *Py. ultimum*, which causes damping-off and root rot on more than 300 diverse hosts, including corn, soybean, wheat and ornamentals [6].

*Pythium* spp. are mostly generalists and non-specific in host range, occurring in habitats ranging from aquatic to (moist) terrestrial. The host range includes insects, fish, mammals or algae, but the majority of *Pythium* spp. are pathogens of plants occurring in water or soil. Some species are of great economic importance in agriculture, horticulture and forestry [2]. Apart from causing damping-off, *Pythium* spp. can infect root systems of older plants, resulting in necrotic lesions [7]. Infected seedlings may survive infection, but vigour is often substantially reduced [1]. Susceptibility of plants significantly decreases with host age; however, older plants are less likely to show signs and symptoms of *Pythium* infection [8].

The international spread of oomycetes is primarily a result of anthropogenic activity. It is recognised that populated areas might receive high amounts of soil-borne pathogen inoculum due to recurrent out-planting of infected ornamental plants, which highlights the potential of human-mediated dispersal of plant pathogens and the difficulties in controlling these problems [9,10]. Several studies have confirmed the international plant trade as one of the most important pathways and threats based on the numbers of alien species introduced [11,12,13,14,15].

Indigenous pathogens tend to cause little damage to native hosts, as the two groups of organisms have co-evolved over millennia [16]. However, when the pathogens are introduced into naïve ecosystems, the lack of host resistance to the invasive organisms can lead to disease outbreaks [11,17] resulting in damage to forest and riparian ecosystems and irreversible economic, social and biological losses [14,18].

In 2008, a previously unknown *Pythium* species was isolated and characterized from soil samples taken at an elevation of 1600 m in the Shivalik Hill Range, Himalayan mountains of the northern Indian state of Jammu and Kashmir [4] and was described as *Pythium kashmirense* sp. nov. Closely related species include *Pythium valencianum* which [4] is considered a homonym of *Py. kashmirense*, *Py. plurisporum*, *Py. periilum*, *Py. inflatum*, *Py. angustatum*, *Py. folliculosum* and *Py. catenulatum*, all in ‘Clade B’, as defined by [19] and [4].

Later, *Py. kashmirense* was isolated during a survey of soya bean crops in North Dakota [20], the first report in the United States. Pathogenicity tests confirmed that this organism could infect and cause damage to soya bean plants. A survey of *Pythium* and *Phytopythium* species was conducted at a hydroponics centre located in São Paulo and nearby cities in Brazil including baiting water from an artesian well, nutrient solution, substrates used for seedling production, and roots of hydroponically grown crops. *Pythium kashmirense* was found following the baiting of samples from root substrates of different crops including *Apium graveolens* L., *Coriandrum sativum* L., *Eruca sativa* L., *Lactuca sativa* L., *Lepidium sativum* L. and *Spinacia oleracea* L. [21]. *Pythium kashmirense* was also found in Iran on cucurbits showing root and crown rot in a field in the Kermanshah province [22].

During work at the University of Aberdeen examining the oomycete diversity associated with ornamental plants sold in garden centres and via internet platforms [15], a *Pythium* species was isolated from *Viburnum plicatum* ‘Lanarth’ obtained from a nursery in the southwest of England. *Viburnum plicatum* is an ornamental woody shrub commonly grown in European gardens. The unknown *Pythium* species formed a chrysanthemum-like pattern on potato dextrose agar (PDA) and had an ITS sequence with 99% congruence with *Py. kashmirense* accessions in GenBank. This was the first report of *Py. kashmirense* in the UK and in Europe.

The aim of the work reported here was to characterize the *Py. kashmirense* isolates from *V. plicatum* further, and develop a better understanding of the host range, pathogenicity and virulence.

## 2. Materials and Methods

### 2.1. Isolation, Identification and Characterization

Between 2014 and 2015 isolates (P0 96 and P097, DNA Code and Accession Number: ABD:133 MF115269 and ABD:134 MF115270, respectively) of *Py. kashmirense* were obtained using soil-baiting techniques from the compost of five plants of *Viburnum plicatum* ‘Lanarth’, purchased from a plant nursery retailer in the UK through the internet; no direct isolations from roots or stems were obtained. Symptoms observed included withered foliage, necrosis and defoliation [15]. Once pure cultures were obtained, the isolate was characterized morphologically and identified by PCR. DNA extraction was performed using a modified version of the method of [23]. The ITS region (ITS1—5.8S—ITS2) was amplified using general primers ITS4 [24] and ITS6 [25]. Each reaction contained 1× PCR Buffer (containing 1.5 mM MgCl_2_), 0.2 mM each dNTP, 0.2 μM each primer, 1 U Taq polymerase (Promega, Chilworth, UK) and 2 μL template DNA. The PCR mix was adjusted to a final volume of 25 μL with water. The program used for amplification of the ITS region was 95 °C for 3 min, 35 cycles of 94 °C for 30 s, 55 °C for 30 s, 72 °C for 1 min. The final extension was carried out at 72 °C for 10 min. PCR products were visualized by electrophoresis on 1.5% agarose gel. Amplified samples were purified with the EZNA Cycle Pure Kit (Omega Bio-Tek, Georgia, USA) and quantified using a Nanodrop ND-100 spectrophotometer (Nanodrop Technologies, Oxford, UK). PCR products were adjusted to the required concentration and sequenced by Source Bioscience Lifesciences (Nottingham, England). Sequencing results were compared with databases in GenBank using the BLAST tool with the algorithm ‘blastn’.

Colony patterns of *Pythium kashmirense* were described from 7-day-old cultures grown at 25 °C in the dark on four different media: PDA; malt extract agar (MEA, 50 g L^−1^, Oxoid, Basingstoke, England); 10% clarified V8A (cV8A); 10% carrot agar (CA). V8 juice was clarified by centrifugation and filtration. One litre of cV8A contained 100 mL clarified V8 Juice, 900 mL distilled water, 2 g CaCO_3_, 15 g agar. 10% CA was prepared using 200 g fresh carrots blended in 500 mL of distilled water and squeezed through cheese cloth. One litre of CA contained: 100 mL carrot juice, 900 mL distilled water, 15 g of agar [26,27,28].

### 2.2. Host Range and Pathogenicity

In vitro and in vivo assays were conducted to evaluate the host range and pathogenicity of *Py. kashmirense* (P096) using healthy germinating seeds of four plant species: *Glycine max* (soya bean); *Phaseolus vulgaris* (common bean); *Lupinus angustifolius* (blue lupin) and *Cucumis sativa* (cucumber). Due to difficulties with long-term processes needed to raise *Viburnum* plants from seeds or cuttings, in vitro inoculation assays were conducted using healthy non-suberized roots, excised from first-year seedlings of *V. opulus* bought from a local nursery.

‘Pathogenicity’ was defined as the ability of the pathogen to cause disease, and ‘virulence’ as the extent of the pathology (disease) caused.

#### 2.2.1. In Vitro Assays—Seed Rot Tests

In vitro assays focused on seed infection, evaluation of germination rates and a range of variables related to seed viability after inoculation, including ‘mortality’, ‘root disease severity’, root length, fresh plant weight and dry plant weight.

##### 2.2.1.1. Preparation of Pre-Germinated Seeds

Soya bean, common bean, blue lupin and cucumber seeds were surface-sterilized in 2.5% sodium hypochlorite solution for five min, washed three times in sterile distilled water and soaked in sterile distilled water overnight in the dark at 25 °C. Imbibed seeds were placed on 2% water agar (WA) in 90 mm diameter, with Petri dishes at room temperature for two days (pre-germination). 

##### 2.2.1.2. Preparation of *Py. kashmirense* Inoculum

The cultures of *Py. kashmirense* were maintained on PDA and periodically grown in tryptone soya broth (TSB) and nutrient broth (NB) to check and avoid bacterial contamination.

An inoculum of *Py. kashmirense* was prepared by placing a 5 mm^2^ agar plug growing on PDA at the centre of a 9 cm Petri dish of 2% WA and incubating at 25 °C in the dark for three days until the culture covered the agar surface. Five pre-germinated seeds were spaced equally in the Petri dish on the culture surface. Controls consisted of five pre-germinated seeds placed on 2% WA without *Py. kashmirense* cultures. Petri dishes were incubated at 25 °C with a 12-h photoperiod at a light intensity of 30–50 µmol/m^2^/s for 7 days. For each plant species, the experiment consisted of 5 replicates for both *Py. kashmirense* and the control, and the experiment was repeated 3 times. 

##### 2.2.1.3. Seed Rot Test Analysis

Seeds were examined after 7 days and the percentage of germinated seeds and the number of dead/alive seeds per Petri dish recorded.

Disease Severity (DS) was assessed by visual evaluation of each seed on the day of harvesting: seeds were rated for presence of rot using a categorical 0–4 disease severity scale modified from published methods [29,30]:

0 = seed germinated without visible infection and symptoms (a seed was defined as germinated when the primary root length equalled the seed length);
1 = seed germinated with reduced growth and light discoloration or lesions;
2 = seed germinated with severely reduced growth and strong visible discoloration and lesions on roots;
3 = seedling died after germination with partial colonization by mycelial growth;
4 = seed died before germination with complete colonization of seed by mycelium.

On the day of harvesting, after evaluation of visible infections and symptoms, the roots were washed in tap water and transferred to the analytical laboratory to proceed with root length, fresh plant weight and dried plant weight measurements. Root length measurements were carried out with electronic digital callipers. Plants were weighed on an analytical balance before and after drying. Plants were dried until constant weight in a Gallenkamp drying oven at 60 °C, generally for 24–48 h.

#### 2.2.2. In Vivo Assays—Damping-Off Tests

Relative susceptibility to *Py. kashmirense* infection of the plant species listed above was assessed using in vivo assays carried out in Magenta boxes (74.79 × 74.79 × 99.08 mm) containing 70 g sterile substrate (All Purpose Growing Medium, Sinclair). Boxes with the growing medium were autoclaved for 60 min twice, at an interval of 24 h.

##### 2.2.2.1. Preparation of Germinated Seeds

Seeds were surface-sterilized, as described for the in vitro assays, and soaked overnight in sterile distilled water before placing on 2% WA in Petri dishes and incubating at 25 °C until germination.

##### 2.2.2.2. Preparation of *Py. kashmirense* Inoculum

The inoculum was prepared following a slightly modified version of the protocol of [31,32]. *Pythium kashmirense* isolates were sub-cultured to fresh PDA and incubated at 25 °C in the dark for 7 days. Six 5 mm^2^ plugs of *Py. kashmirense* were transferred from these cultures to 150 mL glass vessels containing 50 g millet grains and 35 mL 20% unclarified V8 (uV8) broth. Controls used sterile PDA plugs and otherwise were treated in the same way. Prior to use, millet and uV8 broth were autoclaved separately: millet was autoclaved for 60 min twice, with a 24 h interval between runs, whereas the 20% uV8 broth was autoclaved once for 15 min. Cultures were shaken by hand every two days to ensure homogeneous distribution of inoculum growth. The pathogen was left to colonize the millet-uV8 broth medium in the dark at 25 °C for 15 days prior to soil inoculation. 

Plants were grown in Magenta boxes, as described above. A base layer of 50 g sterile substrate was placed in each Magenta box, followed by a layer of 15–20 g *Py. kashmirense* inoculum (or control inoculum), covered with another layer of 20 g sterile substrate and gently mixed with a sterile spoon to ensure homogeneity of the inoculum in the soil. One germinated seed was placed in each Magenta box and incubated at 25 °C with a 12 h photoperiod at a light intensity of 30–50 µmol/m^2^/s for 10 days. Plants were watered every two days until harvesting to ensure a favourable environment for pathogen establishment and development. For each plant species, the experiment consisted of 8 replicates each for *Py. kashmirense* and the controls, and the experiment was repeated twice.

##### 2.2.2.3. Damping-Off Test Analysis

After 10 days of growth, the numbers of emerged/not-emerged plants and alive/dead plants were recorded.

In order to assess disease severity, foliar symptoms and root rot were rated visually using a modified 0–4 disease severity scale [29,30]: 

Foliar symptoms: 0 = foliage without visible infection symptoms; 1 = foliage yellowing; 2 = foliage yellowing and tip wilting; 3 = yellowing, tip and total foliage wilting; 4 = plant death.

Root rot disease severity: 0 = roots without visible infection or discoloration; 1 = roots with light discoloration and light root and stem rot; 2 = short roots with discoloration; 3 = short roots with severe discoloration and root rot; 4 = plant death.

Root length plus fresh and dry weight were also recorded on the day of harvest, as described for the in vitro assays. Roots were dried to constant weight in a Gallenkamp Drying oven at 60 °C, generally for 24–48 h depending on the plant species.

#### 2.2.3. Inoculation of *Viburnum* spp.

Young healthy seedlings of *V. opulus* were bought from a commercial nursery (Christie–Elite Nurseries, Forres, Morayshire, UK) for use in inoculation tests, due to the difficulties and long processes and treatments needed to grow *Viburnum* plants from cuttings or seed. The pathogenicity test protocols were slightly modified and combined with the procedure described by [33].

*Pythium kashmirense* was subcultured to 2% WA in Petri dishes and incubated for 3 days at 25 °C in the dark. Five root segments, 3 cm in length, of *V. opulus* previously washed in running tap water and rinsed in sterile distilled water, were placed in the cultures of *Py. kashmirense* and incubated at 25 °C with a 12 h photoperiod. The experiment consisted of 9 inoculated and 7 control (2% WA with no *Py. kashmirense*) Petri dishes.

A second experiment used *V. opulus* roots submerged in sterile substrate. A *Pythium kashmirense* inoculum was prepared and mixed into the substrate, as described in Section 2.2.2.2. Five root segments of *V. opulus*, cleaned as described above, were buried in the mixed substrate and incubated at 25 °C. The experiment consisted of 8 inoculated and 8 control Magenta boxes.

After 7 days, visual symptoms and disease severity on the cleaned roots were recorded using the 0–4 scale described above for the root rot in vitro assays (Section 2.2.1.3).

#### 2.2.4. Reisolation of the Pathogen

Reisolations were performed three times for each plant species and assay. The original *Py. kashmirense* cultures used for inoculation and the cultures obtained by reisolation from root and seed samples, respectively, were transferred to corn meal agar-PARBP [34], incubated at 22 °C for 4–5 days and identified by morphological comparison.

### 2.3. Data Analysis

Six response variables were recorded in in vitro assays: germination rate; mortality; seed disease severity; root length (mm); fresh weight (g) and dry weight (g). ‘Germination rate’ was the percentage of germinated seeds (a seed was defined as germinated when the primary root length equalled the seed length) in each Petri dish; ‘mortality’ was the percentage of dead or non-germinated seeds. ‘Seed disease severity rating’ was converted to a continuous Disease Severity Index (DSI), using the formula of [30]:

$$\mathrm{DSI}=\frac{\sum \left(\mathrm{severity}\;\mathrm{rating}\times \mathrm{seeds}\;\mathrm{per}\;\mathrm{rating}\right)}{\left(\mathrm{total}\;\mathrm{seeds}\times \mathrm{highest}\;\mathrm{severity}\;\mathrm{rating}\right)}\times 100$$

All variables were considered as continuous in the statistical analyses.

Root length, fresh weight and dry weight were determined for the living, germinated seeds. 

Data were analysed using R (v 3.3.1; R Foundation for Statistical Computing, Vienna, Austria) with an Open Source License.

Normality was examined using the Shapiro–Wilk Normality Test and adjusted by the Holm Method, whereas homogeneity of variances was tested using the Levene Test with the mean as a centre. When both groups (treatment and control) had normal distribution, differences were tested using the Welch Two Sample t-test. When normality could not be assumed, the non-parametric Wilcoxon rank sum test was used. 

In the in vivo experiments, the variables ‘Emerged-Not emerged’ and ‘Mortality’ (Dead-Alive) were binary and a ‘2-sample test for equality of proportions without continuity correction’ (Pearson’s Chi-squared test) was used. Root disease severity and foliar symptoms were considered as qualitative ordinal variables that could not be properly analysed as continuous variables. Thus, data were analysed using the Wilcoxon test to illustrate differences between control and inoculated plants. All other variables from the in vivo experiment (root length, fresh weight, dry weight) were analysed as explained for the in vitro experiment. 

Pathogenicity of *Py. kashmirense* was also compared between plant species using the in vitro and the in vivo assay results. In this case, the groups were the individual plant species inoculated with *Py. kashmirense*, and the comparisons were made as follows. 

For the in vitro assays, comparisons were made using the variables ‘germination rate’, ‘mortality’ and ‘seed disease severity’. First, Normality and Homogeneity of Variances were tested. When those hypotheses could not be assumed, the Kruskall–Wallis Pairwise comparison using the Wilcoxon rank sum test was used. When normality and homogeneity of variances were present, an ANOVA model was used, and, if significant differences were observed, a multiple comparison of means by Tukey Contrasts was applied. If data had a normal distribution but variances were not homogeneous, the Welch Test One-Way Analysis of means not assuming variances and Pairwise comparisons using t-tests with non-pooled SD were used.

For the in vivo assays, comparisons were made using the variables ‘Emerged-Not emerged’, ‘mortality’, ‘Root DS’ and ‘Foliar Symptoms’. A 2-sample test for equality of proportions without continuity correction (Pearson’s Chi-squared test) was used to compare the effects *Py. kashmirense* on the different plant species based on the variables ‘Emerged-Not emerged’ and ‘Mortality’ (Dead-Alive). The other variables (Root DS and Foliar Symptoms) were analysed as detailed above in the in vitro assays. 

In all cases a significance level of *p* < 0.05 was used.

## 3. Results

### 3.1. Isolation, Identification and Characterization

**Figure 1:**
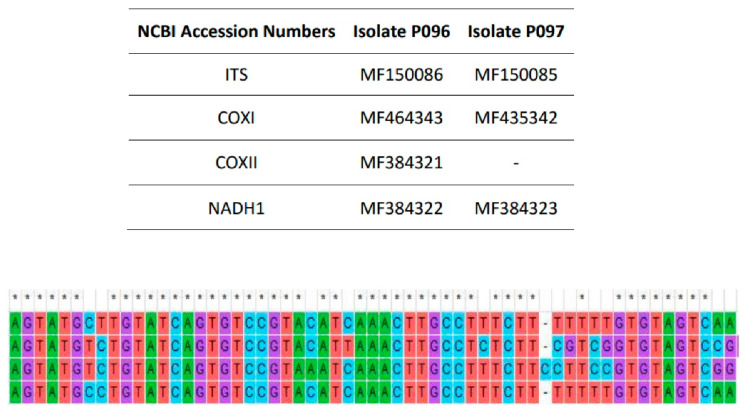
Accession numbers of the sequences of *Pythium kashmirense* deposited in NCBI (Above); Sequence comparison of the ITS region of *Pythium kashmirense* (P096), *Py. rostratifingens* (P216), *Py. sylvaticum* (P194) and *Py. dissotocum* (P107), from top to bottom, sequence fragment from 496 to 554 bp (below). * indicates no variation in the nucleotide at that single position.

All sequences obtained from amplification of the different loci were submitted to NCBI. Figure 1 shows the accession numbers for each isolate and different genes, and the comparison of ITS sequences of *Py. kashmirense* and other *Pythium* species.

**Figure 2:**
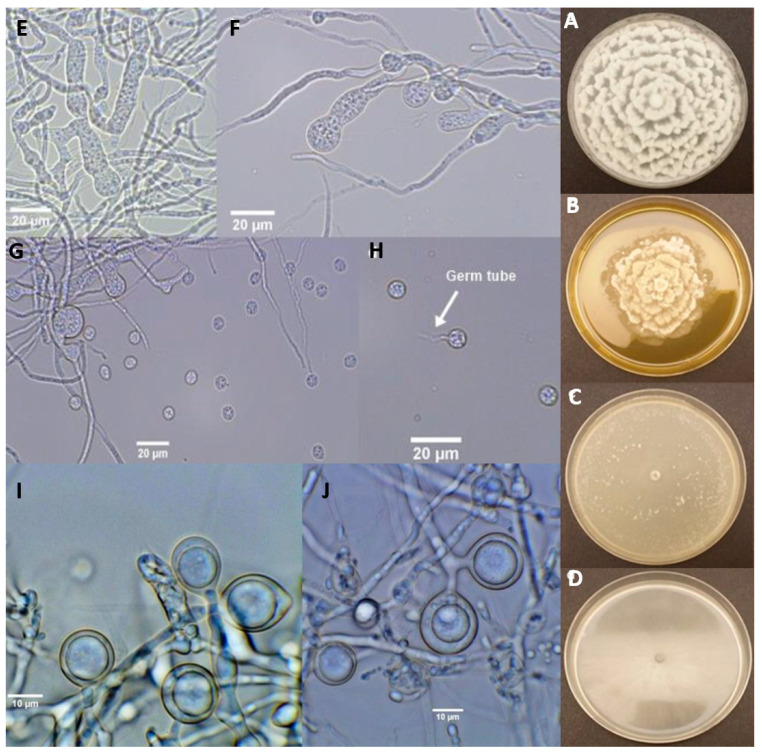
Growth characteristics of *Pythium kashmirense*. Growth on (**A**) potato dextrose agar, (**B**) malt extract agar, (**C**) 10% clarified V8 Agar and (**D**) 10% carrot agar after seven days of incubation at 25 °C in the dark. Asexual structures of *Pythium kashmirense*: (**E**–**G**) large and inflated sporangia and (**G**,**H**) encysted zoospores; (**H**) Encysted zoospore with an emerging germ tube; (**I**,**J**) Sexual structures (immature oogonia with plerotic oospores) of *Pythium kashmirense*.

*Pythium kashmirense* isolates had hyaline mycelium in all media examined. Colonies on MEA showed slower growth in comparison to those on PDA, 10% CA and 10% cV8A, and mycelium appeared clumped with submerged colony edges. A characteristic chrysanthemum growth pattern appeared only on PDA, whereas growth was more dispersed and cottony on 10% cV8 (Figure 2a,b). On 10% CA, colony morphology was completely submerged and lacked any pattern (Figure 1d). 

Isolates of *Py. kashmirense* had filamentous and inflated sporangia, sometimes appearing to be linked to each other (Figure 2a,f,g). Oogonia were observed surrounded by antheridial branches and contained plerotic oospores (Figure 2i,j).

### 3.2. Host Range and Pathogenicity

In vitro and in vivo assays on different plant species (*Glycine max*, *Phaseolus vulgaris*, *Lupinus angustifolius*, *Cucumis sativa*) were conducted to evaluate the host range and pathogenicity of *Py. kashmirense.* For all variables measured, there were differences between inoculated treatments and non-inoculated controls, but not all differences were significant. 

#### 3.2.1. In Vitro Assay—Seed Rot Test

**Figure 3:**
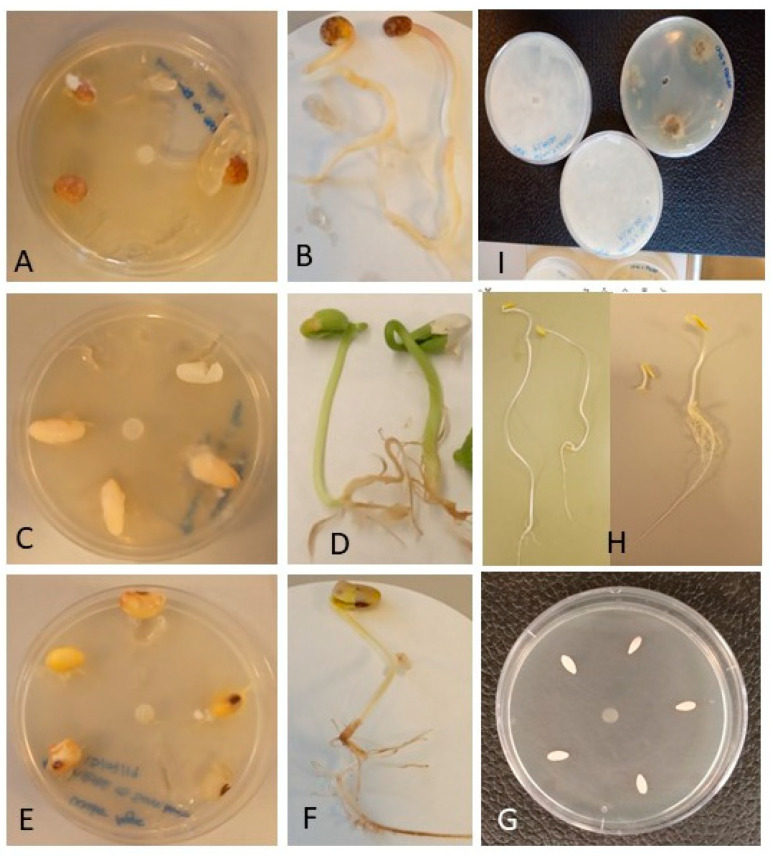
In vitro assay. (**A**) Blue lupin inoculated with *Py. kashmirense*, (**B**) Control of Blue lupin, (**C**) Common bean inoculated with *Py. kashmirense*, (**D***)* Control of Common bean, (**E**) Soya bean inoculated with *Py. kashmirense*, (**F**) Control of Soya bean, (**G**) Cucumber inoculated with *Py. kashmirense.* (**H**) Three controls of cucumber compared with an infected seed. (**I**) Reisolation of *Py. kashmirense* from inoculated roots to fulfil Koch’s postulates.

The germination rates for soya bean, common bean and blue lupin were significantly lower in seeds inoculated with *Py. kashmirense* compared to the controls (*p* < 0.05) (Figure 3, Table 1). The differences in germination rates for cucumber in the inoculated and control treatments were not significantly different. However, for all tested plant species, including cucumber, the mortality and disease severity indices were significantly higher in *Py. kashmirense*-inoculated seeds than in controls (*p* < 0.05).

Roots were significantly shorter in *Py. kashmirense*-inoculated plants of blue lupin, cucumber and common bean (*p* < 0.05) compared to controls, but no significant difference was observed for inoculated soya bean roots. *Pythium kashmirense* inoculation also had a significant negative impact on the fresh weight of blue lupin and cucumber plants compared to controls (*p* < 0.05), but no significant difference was observed for soya bean or common bean compared to the respective controls (Table 1).

#### 3.2.2. In Vivo Assay—Damping-Off Test

**Figure 4:**
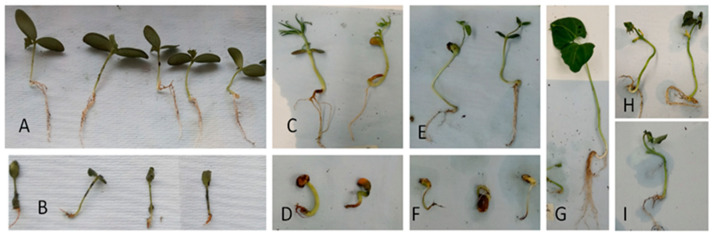
Pathogenicity indices of four plant species inoculated with *Py. kashmirense*. In vivo assay. (**A**) Cucumber-control, (**B**) Cucumber-inoculated, (**C**) Blue lupin-control, (**D**) Blue lupin-inoculated, (**E**) Soya bean-control, (**F**) Soya bean-inoculated, (**G**) Common bean-control, (**H,I**) Common bean-inoculated.

Significant pathogenicity of *Py. kashmirense* occurred on all inoculated plants: soya bean, common bean, blue lupin and cucumber (*p* < 0.05) (Figure 4, Table 2). Fewer numbers of plants emerged in the inoculated treatments compared with the controls, approximately half for soya bean and blue lupin. Mortality (proportion of dead plants), foliar symptoms and root disease severity were significantly higher in all *Py. kashmirense*-inoculated plants compared to control plants. Differences were particularly marked when assessing the mortality of soya bean and blue lupin, with approximately 70% greater death in inoculated plants. Similarly, foliar symptoms and root disease severity were significantly different for all plant species tested, with up to ten-fold differences in inoculated plants compared with controls (Figure 4, Table 2). In addition to damping-off traits, symptoms on inoculated plants included poor vigour, seed rot, yellowing leaves, decay and the presence of brownish lesions. 

Root length, fresh weight and dry weight were also lower in inoculated plants compared to the controls (Table 2). Root length for all plants was significantly lower compared to the controls (*p* < 0.05), whereas fresh and dry weights in comparison to controls were significantly lower only for common bean and cucumber (Table 2).

#### 3.2.3. Relative Susceptibility of Four Plant Species to *Py. kashmirense*

**Figure 5:**
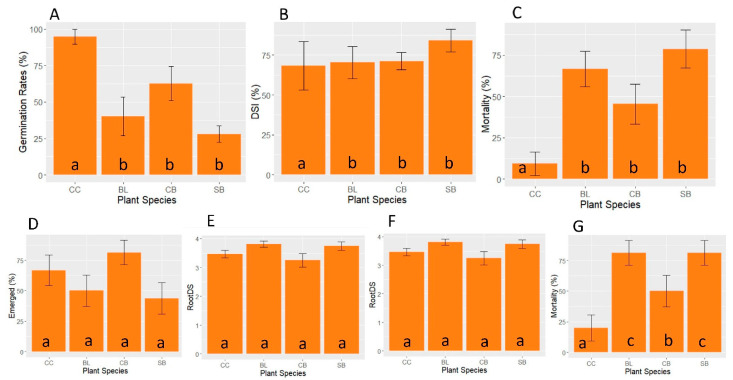
Pathogenicity indices of four plant species inoculated with *Py. kashmirense*. In vitro inoculation of cucumber (CC) blue lupin (BL), common bean (CB) and soya bean (SB) with *Py. kashmirense* (**A**) Germination rate, (**B**) Disease Severity Index (DSI) and (**C**) Mortality. In vivo inoculation of cucumber (CC), blue lupin (BL), common bean (CB) and soya bean (SB) with *Py. kashmirense*; (**D**) Emerged rates; (**E**) Foliar Symptoms; (**F**) Root disease severity index (DSI); (**G**) Mortality. The letters indicate the comparisons that resulted as significant after the statistical analyses tests.

To assess relative pathogenicity, cucumber, blue lupin, common bean and soya bean were inoculated in vitro with *Py. kashmirense*, and assessed for seed germination rate, disease severity and mortality; in addition, a multiple comparison test between plant species was carried out (Figure 5A–C). Seed germination rate and mortality differed significantly between the plant species (Kruskal–Wallis test, germination *p* < 0.001; mortality *p* < 0.001). A multiple comparison showed that, for both germination rates and mortality, cucumber was significantly less susceptible to *Py. kashmirense* than soya bean, common bean or blue lupin. Cucumber, blue lupin, common bean and soya bean were inoculated in vivo with *Py. kashmirense*, assessed and compared for emergence rates, foliar symptoms, root disease severity and mortality (Figure 5D–G). Although no significant differences were found for the proportion of emerged plants, foliar symptoms or root disease severity, the number of dead plants differed significantly between plant species (Chi-squared test, *p* < 0.001). Most (81.2%) soya bean and blue lupin died, in contrast to 20% and 50% of cucumber and common bean plants, respectively.

### 3.3. Inoculation of Viburnum Opulus Roots

Inoculation of *Viburnum opulus* roots with *Py. kashmirense* caused severe necrosis and complete decay. In addition, there was a characteristic sulphurous smell produced by the *Py. kashmirense* infection.

**Figure 6:**
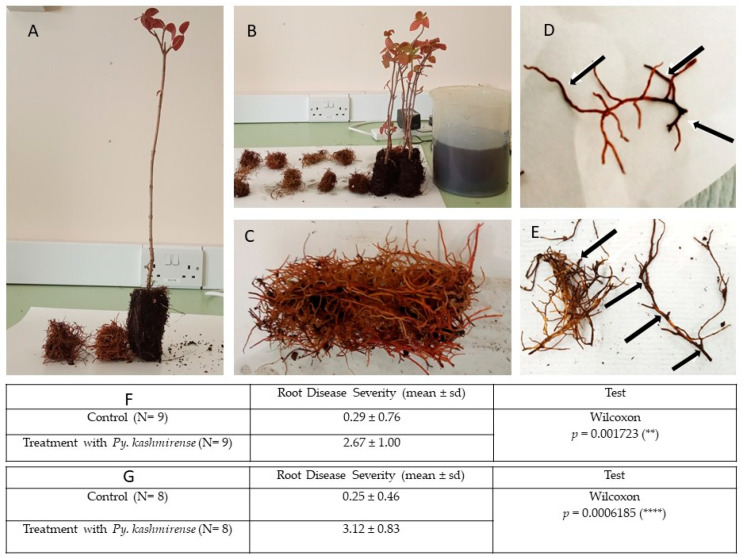
Inoculation of *Viburnum opulus* roots with *Py. kashmirense*. (**A**,**B**) Healthy seedlings of *Viburnum opulus*; (**C**) Healthy root system of *Viburnum opulus* cleaned with sterile distilled water; (**D**,**E**) *Viburnum opulus* roots after inoculation with *Pythium kashmirense* in vitro, showing discoloration and necrosis on around the 40 to 50% of the root segments; (**F**) Data analysis of *Viburnum opulus* Root Disease Severity in the in vitro assay; (**G**) Data analysis of *Viburnum opulus* Root Disease Severity in the in vivo assay; (**D**,**E**) *Viburnum opulus* roots after inoculation with *Pythium kashmirense* in vitro, showing discoloration and necrosis on around the 40 to 50% of the root segments; (**F**) Data analysis of *Viburnum opulus* Root Disease Severity in the in vitro assay; (**G**) Data analysis of *Viburnum opulus* Root Disease Severity in the in vivo assay. ** means *p* ≤ 0.01, **** means *p* ≤ 0.0001.

In the in vitro assay, the mean root disease severity was 2.67 for the *Py. kashmirense*-inoculated treatment, compared with 0.23 in controls. Similar results were obtained in the in vivo assay (Mean RDS: 3.13 inoculated; 0.25 control). A Wilcoxon test showed that differences between inoculated roots and controls were significant (*p* = 0.002 and *p* < 0.001) for in vitro and in vivo assays, respectively) (Figure 6).

## 4. Discussion

The major aim of this work was to increase our understanding and knowledge of the biology and host range of of *Py. kashmirense* in order to develop management protocols.

In vivo assays (Figure 4) demonstrated clear evidence of pathogenicity symptoms on all plants inoculated with *Py. kashmirense.* The proportion of soya bean and blue lupin plants that emerged above the compost was less than half in the presence of *Py. kashmirense*; the difference in pathogenicity (proportion of dead plants), foliar symptoms and root disease severity for the inoculated plants were even greater in some cases. Root lengths were also considerably shorter, while differences in fresh and dry weight were less clear; fresh and dry weights, however, were on average always lower in the inoculated groups; however, the results were not conclusive for all plant species. These results concur with those observed in the in vitro experiments (Figure 3) and suggest that, if an infected plant survived, it was not clear that future development would be strongly affected by the infection; thus, it cannot be stated that the *Py. kashmirense* had a fitness cost to the surviving plants, ultimately affecting their productivity and quality.

Regarding the relative pathogenicity of *Py. kashmirense* to different plant species, conclusive differences were found in vitro in terms of germination rates and pathogenicity, suggesting that cucumber (Cucurbitaceae) was less susceptible to *Py. kashmirense* than the other tested plant species (all Leguminoseae). In contrast, the in vivo experiments did not show remarkable differences in the proportion of emerging plants, foliar symptoms or root disease severity. There was, however, lower mortality in the cucumber and common bean, compared with lupin and soya bean.

Although many *Pythium* spp. are generalists and non-host-specific in range, these results indicate that there may be variations in virulence between certain plant families. The results in Figure 3 and Figure 4 support the findings of [20] demonstrating the ability of *Py. kashmirense* to cause damage on soya bean in North Dakota, and add information about other potential hosts in the UK and globally. These findings increase understanding of the potential for damage caused by *Py. kashmirense* should it become more widespread in the environment. Moreover, as [20] observed, *Py. kashmirense* shows differential virulence on different plants species.

In future work, however, the virulence tests with *Py. kashmirense* must be widened to include more, unrelated host species to gain greater insight into host range. Moreover, the potential virulence on the host from which the isolates of the pathogen were obtained in this work remains unclear: *Py. kashmirense* inoculation clearly caused damage to roots of *V. opulus* when compared with control roots, but the defensive capacities of the roots were diminished by severing from the mother plants. Difficulties in obtaining the required numbers of *Viburnum* plants without oomycetes on the roots systems led to the use of surface-sterilized root systems from the readily available *V. opulus*, but ideally inoculations should be made on the same variety or others of *V. plicatum*. It also remains to be seen whether the pathogen shows differential virulence on varieties of the currently known hosts.

This work supports previous studies demonstrating that many *Pythium* species cause damping-off and root rot in several important agricultural crops and also on ornamentals and forest plants in both nurseries and when planted in the field, especially in warm and wet conditions [1,2,7,35]. Species are adapted to different climates, including temperate, tropical and subtropical; edaphic water availability has a critical role in the development of infections. Many reports have described isolating and identifying *Pythium* species present in agricultural crops. For example, [29] isolated eleven species, and identified two distinct morphological groups of *Pythium*, of which six species were moderately to highly pathogenic on corn (*Zea mays*) seed and nine species highly pathogenic on soybean seed. In northwest Iran, 78 *Pythium* isolates were obtained from diseased sugarbeet seedlings that were planted in soil samples collected from different sugarbeet fields in the country; *P. ultimum* var. *ultimum* was the dominant species, and *P. aphanidermatum*, *P. deliens*, *P. oligandrum*, *P. ostracodes*, *Pythium* group HS and *Pythium* group T were other species identified [36]; during the greenhouse pathogenicity test, a wide range of *Pythium* spp. isolated in the work was tested, demonstrating that three species were non-pathogenic, whereas four isolates of *Py. aphanidermatum*, *Py. ultimum* var. *ultimum* and *Pythium* group HS were very highly pathogenic. The remaining 24 isolates tested were weakly, moderately or highly pathogenic. A further demonstration of the diversity of *Pythium* spp. that may be found within a restricted area was given by [37], who characterized 27 species in the genus from soil samples taken in 12 corn-soybean rotation fields in Illinois; an in vitro pathogenicity assay on soybean seedlings showed that *Py. cryptoirregulare*, *Py. irregulare*, *Py. sylvaticum*, *Py. ultimum* var. *sporangiiferum* and *Py. ultimum* var. *ultimum* were highly pathogenic on soybean seedlings. More recently, the use of real-time PCR (TaqMan) analyses of the compost used for raising ornamental woody plants clearly showed the high diversity of oomycota, with many species of *Phytophthora*, *Pythium* (including *Py. kashmirense*) and *Phytopythium* that were transported in the 99 plants analysed [15], presenting unequivocal evidence for the phytosanitary dangers posed by this system of trade.

In the last 30–40 years, the horticultural industry has made continuous improvements to shipment and packaging techniques for live plants, which, together with the wide use of internet commerce platforms, have transformed international trade in plants [38]; the increased efficiency of transport is aggravating the risk of the spread of plant pathogens, especially of potted ornamental nursery stock, currently considered the most important pathway of invasion given the high numbers of alien species reported to be introduced [11,12,14]. Numerous *Phytophthora* and *Pythium* species outbreaks have occurred in Europe due to the introduction of contaminated ornamental plants [12,14].

Future work should include inoculations of *Py. kashmirense* on a wider range of important crop species under environmental conditions closer to those in the field and on other ornamental plants. In addition, the effects of different biotic and abiotic factors on disease development should be examined in order to increase understanding of the biology and ecology of the organism. The environmental conditions required for the pathogen to spread and infect should be evaluated, allowing control and management practices to be developed for the disease. It is also possible that different isolates of *Py. kashmirense* vary in aggressiveness, depending on host and climatic conditions.

## Figures and Tables

**Table 1:** In vitro assay data analysis: Germination rates, pathogenicity, disease severity index, root lengths and fresh and dry weights for control and inoculated pre-germinated seeds of plant species. Data are mean ± SD for each factor, with inoculated data in bold, the test used and the *p*-value. NS means *p* > 0.05, * means *p* ≤ 0.05, ** means *p* ≤ 0.01, *** means *p* ≤ 0.001, **** means *p* ≤ 0.0001.

	Germination Rates (%)	Mortality (%)	DSI	Root Length (mm)	Fresh Weight (g)	Dry Weight (g)
Soya Bean N = 15 plates	52 ±2 1.2 **28 ± 10.2** Wilcoxon *p* = 0.0009 (***)	38.6 ± 23.2 **78.6 ± 20.6** Wilcoxon *p* = 5.253 × 10^−5^ (****)	46 ± 23.46 **84 ± 12.84** Wilcoxon *p* = 1.782 × 10^−5^ (****)	59.52 ± 23.88 **59.37 ± 36.84** Welch *p* = 0.9881 (NS)	0.69 ± 0.19 **0.83 ± 0.28** Wilcoxon *p* = 0.0917 (NS)	0.89 ± 0.42 **0.75 ± 0.30** Wilcoxon *p* = 0.2617 (NS)
Common Bean N = 15 plates	84± 23 **62.6 ± 21.2** Wilcoxon *p* = 0.00364 (**)	18.6 ± 24.4 **45.2 ± 22** Wilcoxon *p* = 0.00204 (**)	20.67 ± 23.67 **71.22 ± 9.87** Wilcoxon *p* = 1.549 × 10^−5^ (****)	62.15 ± 31.92 **54.28 ± 30.23** Wilcoxon *p* = 0.197 (NS)	1.34 ± 0.51 **1.14 ± 0.38** Wilcoxon *p* = 0.0629 (NS)	0.64 ± 0.18 **0.71 ± 0.30** Wilcoxon *p* = 0.6632 (NS)
Blue Lupin N = 15 plates	54.6 ± 20.6 **40 ± 23.8** Welch *p* = 0.0416 (*)	52 ± 23.6 **66.6 ± 19.6** Wilcoxon *p* = 0.03385 (*)	43.67 ± 21.08 **70.33 ± 17.97** Welch *p* = 0.0004 (***)	74.06 ± 30.04 **48.87 ± 22.67** Wilcoxon *p* = 0.00036 (***)	1.24 ± 0.37 **1.03 ± 0.29** Welch *p* = 0.0133 (*)	0.95 ± 0.46 **0.92 ± 0.34** Wilcoxon *p* = 0.65 (NS)
Cucumber N = 15 plates	98.6 ± 5.2 **94.6 ± 9.2** Wilcoxon *p* = 0.07885 (NS)	1.4 ± 5.2 **9.4 ± 12.8** Wilcoxon *p* = 0.0173 (*)	0.13 ± 0.35 **68.33 ± 27.49** Wilcoxon *p* = 5.577 × 10^−7^ (****)	122.14 ± 36.28 **87.50 ± 33.27** Wilcoxon *p* = 4.606 × 10^−8^ (****)	0.37 ± 0.10 **0.30 ± 0.10** Wilcoxon *p* = 6.227 × 10^−6^ (****)	0.2 ± 0.003 **0.20 ± 0.02** Wilcoxon *p* = 0.6781 (NS)

**Table 2:** In vivo tests comparing seedling emergence, pathogenicity, foliar symptoms, root disease severity index, root lengths and fresh and dry weights for soya bean, common bean, blue lupin and cucumber seed inoculated with *Py. kashmirense* (rows in bold) compared with controls. Data are mean ± SD for each factor and the *p*-value (for emerged and pathogenicity, sample proportions are shown instead of the mean and SD). NS means *p* > 0.05, * means *p* ≤ 0.05, ** means *p* ≤ 0.01, *** means *p* ≤ 0.001, **** means *p* ≤ 0.0001.

	Emerged (%)	Mortality (%)	Foliar Symptoms (0–4)	RootDS (0–4)	Root Length (mm)	Fresh Weight (g)	Dry Weight (g)
Soya Bean N = 16	93.8 **43.8** X-squared *p* = 0.0023 (**)	6.2 **81.2** X-squared *p* = 1.901 × 10^−5^ (****)	0.38 ± 1.02 **3.8 ± 0.40** Wilcoxon *p* = 6.941 × 10^−7^ (****)	0.38 ± 1.02 **3.75 ± 0.58** Wilcoxon *p* = 6.996 × 10^−7^ (****)	80.24 ± 39.86 **26.36 ± 11.21** Welch *p* = 0.00067 (****)	1.13 ± 0.41 **0.725 ± 0.40** Welch *p* = 0.2058 (NS)	0.17 ± 0.06 **0.14 ± 0.02** Welch *p* = *p* 0.1458 (NS)
Common Bean N = 16	100.0 **81.2** X-squared *p* = 0.0689 (NS)	12.5 **50.0** X-squared *p* = 0.0221 (*)	0.50 ± 1.37 **3.31 ± 0.87** Wilcoxon *p* = 1.602 × 10^−5^ (****)	0.50 ± 1.37 **3.25 ± 0.93** Wilcoxon *p* = 1.645 × 10^−5^ (****)	132.49 ± 46.6 **44.17 ± 31.97** Wilcoxon *p* = 0.00028 (****)	2.49 ± 0.92 **0.98 ± 0.30** Wilcoxon *p* = 1.251 × 10^−5^ (****)	0.23 ± 0.08 **0.11 ± 0.06** Welch *p* = 0.00081 (****)
Blue Lupin N = 16	100 **50** X-squared *p* = 0.0011 (***)	12.5 **81.2** X-squared *p* = 9.751 × 10^−5^ (****)	0.56 ± 1.36 **3.81 ± 0.40** Wilcoxon *p* = 9.751 × 10^−5^ (****)	0.56 ± 1.36 **3.81 ± 0.40** Wilcoxon *p* = 3.582 × 10^−6^ (****)	64.45 ± 31.27 **22.62 ± 10.52** Welch *p* = 0.0023 (**)	1.14 ± 0.22 **0.91 ± 0.52** Wilcoxon *p* = 0.4393 (NS)	0.16 ± 0.04 **0.14 ± 0.02** Welch *p* = 0.434 (NS)
Cucumber N = 16	100.0 **68.8** X-squared *p* = 0.0149 (*)	0 **25** X-squared *p* = 0.0325 (*)	0.06 ± 0.25 **3.5 ± 0.51** Wilcoxon *p* = 0.0325 (*)	0.06 ± 0.25 **3.50 ± 0.51** Wilcoxon *p* = 1.221 × 10^−7^ (****)	94.37 ± 10.69 **31.58 ± 3.90** Welch *p* = 1.791 × 10^−5^ (****)	0.44 ± 0.12 **0.13 ± 0.03** Wilcoxon *p* = 9.909 × 10^−5^ (****)	0.04 ± 0.04 **0.02 ± 0.01** Wilcoxon *p* = 0.00029 (****)
